# Do Chinese cavefish show intraspecific variability in morphological traits?

**DOI:** 10.1002/ece3.6495

**Published:** 2020-07-06

**Authors:** Enrico Lunghi, Yahui Zhao

**Affiliations:** ^1^ Key Laboratory of the Zoological Systematics and Evolution Institute of Zoology Chinese Academy of Sciences Beijing China; ^2^ Museo di Storia Naturale dell'Università degli Studi di Firenze Museo “La Specola” Firenze Italia

**Keywords:** adaptation, cave biology, China, groundwater, stygofauna, subterranean environment

## Abstract

Cavefishes represent one of the most bizarre and intriguing life forms inhabiting groundwater environments. One‐third of the known cavefishes worldwide is endemic to China, and almost half of those belongs to a single genus, *Sinocyclocheilus* (Cypriniformes: Cyprinidae). Analyzing the morphometrics of three *Sinocyclocheilus* species, we aimed to assess whether variability among conspecific populations exists. We predict that populations inhabiting different subterranean habitats (shallow vs. deep) show divergences in specific morphological traits to better cope with the local ecological conditions. Our results showed that the populations showing bigger eyes and reduced humpback were those occurring close to the cave entrance (habitats with light and high food availability), while specimens with smaller eyes and increased humpback were collected from deeper groundwater areas (habitats laying in darkness with food scarcity). This explorative study paves the way for further researches aiming to collect novel data on Chinese cavefishes and highlights the usefulness of these species in evolutionary studies.

## Graphical abstract

Chinese cavefish are among the less studied vertebrates species. Conspecific populations show variability for some morphological traits, which are related to the ecological features of the inhabited environment.

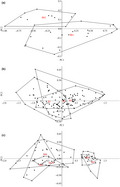

## 1 INTRODUCTION

Cavefishes, with more than 450 known species, are the largest group of vertebrates with adaptations to live in groundwater environments (Ma, Zhao, & Yang, 2019; Niemiller et al., 2019). Although their undeniable importance, studies on groundwater environments and related species were generally lacking until few decades ago (Culver, Kane, & Fong, 1995; Manenti et al., 2018; Smith et al., 2016), probably as the result of the poor awareness toward these animals combined with an objective difficulty of exploration (Buzzacott, Zeigler, Denoble, & Vann, 2009; Gibert, Stanford, Dole‐Olivier, & Ward, 1994). Groundwater environments, like the rest of the subterranean realm, are generally characterized by specific conditions: They lack light, have relatively stable ecological conditions, and are food‐deprived (Culver & Pipan, 2019; Gibert et al., 1994). These conditions are mainly found in the deepest parts, where the external influences are generally absent (Culver & Pipan, 2019); conversely, in the shallowest areas the ecological conditions mostly resemble those found in surface environments (Culver & Pipan, 2014; Lunghi, Manenti, & Ficetola, 2015). This particular circumstance creates a natural ecological gradient going from the connection with surface (light, environmental variability, food availability) to the deepest areas (darkness, environmental stability, food scarcity) (Mammola, 2019).

**FIGURE 1:**
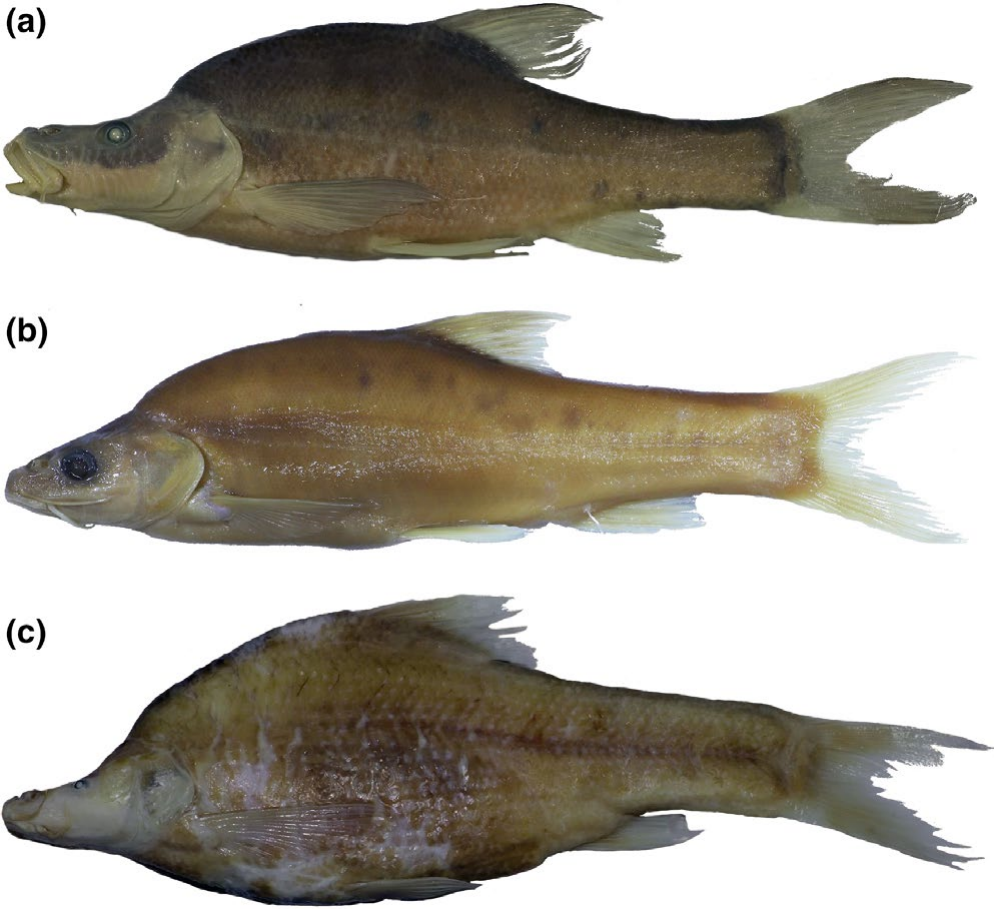
The three species analyzed here: (a) *Sinocyclocheilus brevibarbatus*, (b) *S. jii*; (c) *S. microphthalmus* (modified from Lunghi, Zhao, et al., 2019)

Species inhabiting the deepest subterranean environments often develop similar morphological traits as a consequence of the specific ecological pressures (Armbruster, Niemiller, & Hart, 2016; Culver & Pipan, 2019). One of the most evident characteristic is the reduction (or absence) of eyes, an organ which is useless in environments characterized by permanent darkness (Moran, Softley, & Warrant, 2015; Rétaux & Casane, 2013); to compensate the lack of sight species increase the use of other sensory organs (Bibliowicz et al., 2013; Hyacinthe, Attia, & Rétaux, 2019; Montgomery, Coombs, & Baker, 2001). Species from the deepest subterranean areas are also able to withstand long starvation periods, as local food resources are scarce and inconsistent (Culver & Pipan, 2019; Hervant, 2012). Indeed, cave‐adapted species not only tend to waste less energies (Hervant, 2012), but also try to increase the amount of energy stored in their body (Fišer, 2019; Ma et al., 2019). For this purpose, some species of cavefish can develop a specific morphological adaptation: the humpback (Zhao & Zhang, 2009). This adipose tissue is located on the fish back right behind its head (see Figure 1 in Lunghi, Zhao, Sun, & Zhao, 2019), and it servesas energy storage (Vandel, 1965; Zhao & Zhang, 2009).

China holds more than one‐third of the known cavefish species worldwide, and most of them (>46%) belongs to the genus *Sinocyclocheilus* (Cypriniformes: Cyprinidae), which is, overall, the largest genus of cavefish and is endemic to China (Zhao & Zhang, 2009). In this study, we assessed whether intraspecific morphological variability in three *Sinocyclocheilus* cavefishes exists. Considering the high adaptability characterizing Chinese cavefishes (Fenolio, Zhao, Niemiller, & Stout, 2013; Ma et al., 2019), we hypothesized that conspecific populations inhabiting different habitats may diverge in some morphological traits as a result of a different ecological pressures (Parzefall, 2001). Specifically, we predict that populations inhabiting the deepest groundwater habitats (i.e., area laying in darkness with food scarcity) show smaller eyes and larger humpbacks compared to those living close to the cave entrance (i.e., more illuminated areas with higher food availability). Testing this hypothesis will provide a better understanding of the evolutionary processes behind species adaptation to subterranean environments. Indeed, it is not clear yet to which extent the similarity in morphological traits occurring between cave species is due to a common phylogenetic origin rather than to similar ecological pressures (Culver & Pipan, 2015; Howarth, 2019). Therefore, a potential divergence among conspecific populations may strength the hypothesis supporting a preponderant effect of the local ecological pressures.

## 2 METHODS

### 2.1 Analyzed data

**FIGURE 2:**
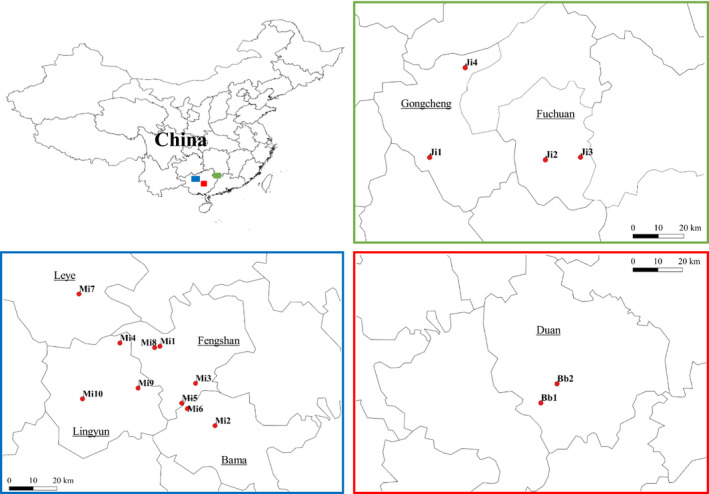
Map of the study area. In each panel (red = *Sinocyclocheilus brevibarbatus*; green = *S. jii*; blue = *S. microphthalmus*), symbols indicate the location of the relative population. Counties are labeled with underscore. Detailed information on collection sites is omitted due to conservation issues and poaching (Lunghi, Corti, Manenti, & Ficetola, 2019)

We analyzed the data published by Lunghi, Zhao, et al. (2019). We focused our study only on three species (*Sinocyclocheilus brevibarbatus*, *S. jii,* and *S. microphthalmus*; Figure 1) as they were those showing a numerousness (≥2 populations with at least 10 individuals) allowing robust analyses: *S. brevibarbatus*, 31 specimens from two populations (Bb1 *N* = 20, Bb2 *N* = 11); *S. jii*, 131 specimens from three populations (Ji1 *N* = 39, Ji2 *N* = 49, Ji3 *N* = 43); *S. microphthalmus*, 78 specimens from five populations (Mi2 *N* = 19, Mi5 *N* = 10, Mi6 *N* = 28, Mi8 *N* = 10, Mi10 *N* = 11) (Figure 2). The dataset provides measurements of 28 fish body parts (Lunghi, Zhao, et al., 2019; but see also Appendix S1), for *S. jii* 27 because the eyeball diameter equals that of the eye (Zhao & Zhang, 2009). Specimens' preservation was not always optimal, and sometimes, the fish body was damaged; the highest rate of damage occurred in fins (>82%).

### 2.2 Statistical analyses

We used the principal component analysis (PCA) to explore the morphological data from the three *Sinocyclocheilus* species. The PCA helps in reducing the group of correlated variables (the cavefish morphometrics) into a set of linearly independent variables. The obtained variables (the principal components) are ranked according to the amount of variance they explain; the first usually explains the largest amount of variance. Data related to fish fins were excluded from the analysis as they were often damaged; this allows to analyze the highest number of specimens. PCA analysis was run for each species singularly and included 21 variables for *S. brevibarbatus* and *S. microphthalmus*, while only 20 for *S. jii* (see Appendix S1). All data were log‐transformed to improve normality and reduce skewness. For each of the first two components (those explaining the highest amount of variance), the significance of the most important correlated variables (loading value ≥ 0.55) was assessed using the analysis of variance (ANOVA) or the multivariate analysis of variance (MANOVA).

The potential inconsistency of the food resources in subterranean environments may affect our analysis, as the humpback area of cavefishes strongly depends by the temporal availability of resources (Vandel, 1965). Indeed, food resources in groundwater environments are irregularly enriched through the seasonal supply of organic matters brought by the incoming water from the surface (Culver & Pipan, 2019). To account for this potential bias, we repeated the analysis for *S. microphthalmus* two more times; the first only considered specimens collected during a single season (March–July) while in the second from a single year (2019).

Analyses were performed using the software PAST and R (R Development Core Team, 2018).

## 3 RESULTS

### 3.1 *Sinocyclocheilus brevibarbatus*

**FIGURE 3:**
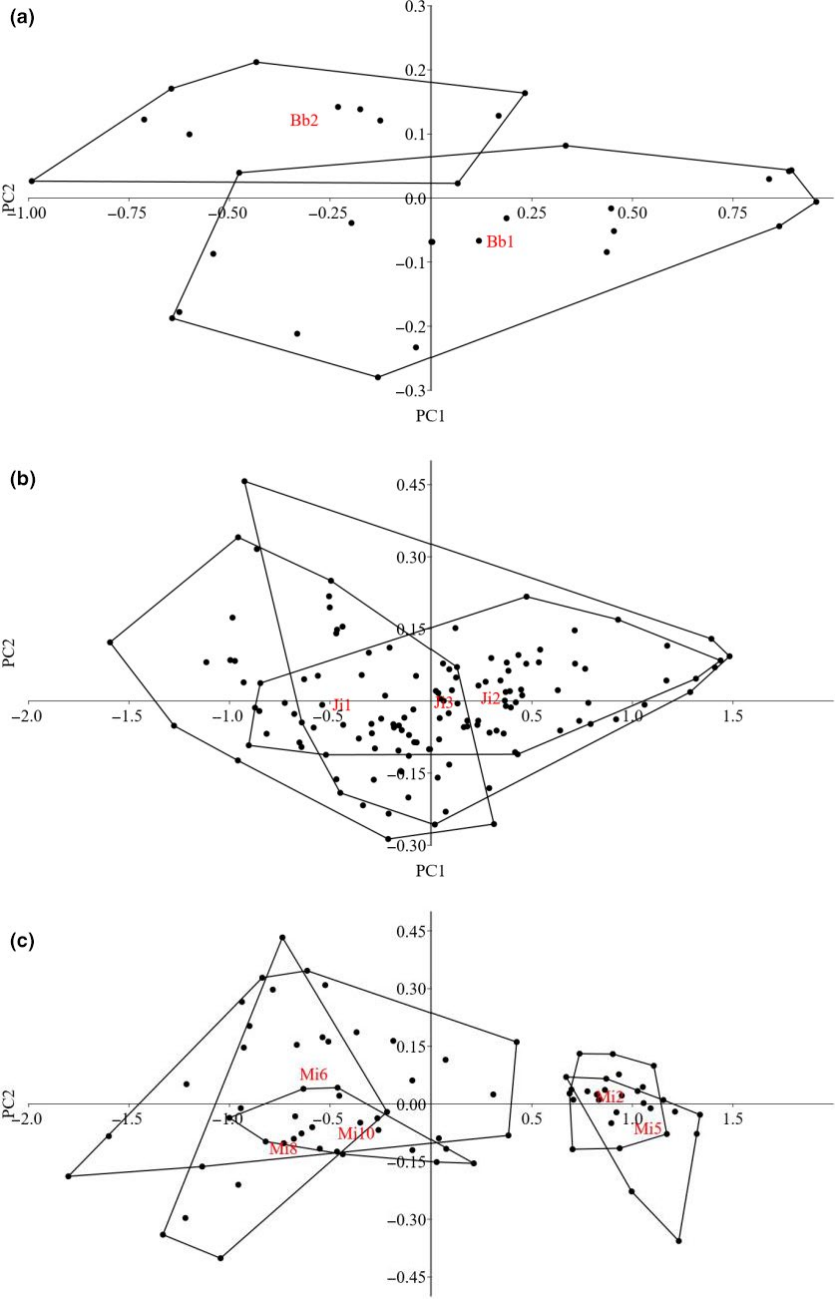
Principal component analysis on the morphology of the three Chinese cavefish species. Scatterplot of the first two axes (PC1 and PC2) showing morphological divergences between the studied populations of (a) *Sinocyclocheilus brevibarbatus*, (b) *S. jii*, and (c) *S. microphthalmus*. The red labels within the convex polygons indicate the relative population

The PCA analysis identified two groups of specimens with little overlap (Figure 3a); the first two components together explained 91.57% of the variance (Table 1). The most important variables for the principal components were the humpback area (PC1) and the eyeball diameter (PC2) (Table 2). Both variables were significantly different between the two populations (eyeball diameter, *df* = 1, *F* = 19.12, *p* < .001; humpback area, *df* = 1, *F* = 8.35, *p* = .007); the Bb1 population included specimens with bigger eyes and smaller humpback area.

**TABLE 1:** List of principal components related to cavefish morphology

	Species									
	*S. brevibarbatus*		*S. jii*		*S. microphthalmus*		*S. microphthalmus*		*S. microphthalmus*	
							Single season		Single year	
Principal Component	Eigen	% var	Eigen	% var	Eigen	% var	Eigen	% var	Eigen	% var
PC1	0.295	86.808	0.377	89.824	0.709	91.765	0.627	90.699	0.692	91.391
PC2	0.016	4.762	0.014	3.343	0.024	3.154	0.027	3.837	0.028	3.697
PC3	0.007	2.192	0.005	1.300	0.010	1.353	0.010	1.402	0.011	1.446
PC4	0.006	1.841	0.005	1.210	0.006	0.833	0.006	0.822	0.005	0.667
PC5	0.005	1.375	0.003	0.831	0.005	0.642	0.005	0.689	0.005	0.643

Note

We report the first five principal components along their eigenvalues and % of variance for each analyzed *Sinocyclocheilus* species and, for the two reduced subsets for *S. microphthalmus*

**TABLE 2:** Contribution of the morphological variables to the first two PCA components

	Species									
	*S. brevibarbatus*		*S. jii*		*S. microphthalmus*		*S. microphthalmus*		*S. microphthalmus*	
							Single season		Single year	
Variables	PC1	PC2	PC1	PC2	PC1	PC2	PC1	PC2	PC1	PC2
Eye_diameter	0.156	0.482	NA	NA	0.097	0.268	0.132	0.180	0.131	0.231
Eye_ball	0.011	0.622^a^	0.121	−0.011	0.116	0.659^a^	0.123	0.568^a^	0.124	0.552^a^
Snout_length	0.174	−0.029	0.181	0.216	0.150	0.068	0.159	0.119	0.159	0.097
Mouth_width	0.161	0.131	0.200	0.269	0.174	0.103	0.177	0.075	0.175	0.066
Lower_jaw_length	0.223	−0.318	0.153	0.138	0.183	−0.071	0.180	−0.077	0.185	−0.060
AD	0.159	0.091	0.158	0.099	0.145	0.021	0.152	0.021	0.151	0.036
B_height	0.102	0.383	0.182	0.121	0.162	0.162	0.157	0.175	0.157	0.192
C_height	0.152	0.113	0.162	0.065	0.169	0.089	0.174	0.093	0.172	0.096
D_height	0.161	0.113	0.164	0.107	0.159	0.051	0.162	0.059	0.160	0.066
DI	0.207	0.047	0.204	0.051	0.206	−0.015	0.207	0.000	0.206	−0.008
AE	0.168	0.080	0.167	0.144	0.151	0.020	0.157	0.023	0.155	0.032
FG	0.192	0.075	0.186	0.221	0.215	0.161	0.200	0.187	0.201	0.184
IK	0.180	−0.037	0.177	0.241	0.208	0.112	0.202	0.155	0.202	0.145
I_depth	0.228	−0.038	0.196	0.055	0.231	0.056	0.226	0.110	0.226	0.101
JW	0.166	−0.016	0.178	0.275	0.226	0.102	0.236	0.090	0.241	0.079
K_depth	0.225	−0.078	0.203	0.050	0.231	0.068	0.229	0.111	0.229	0.098
NO	0.177	−0.187	0.188	0.130	0.187	0.126	0.180	0.101	0.181	0.086
O_depth	0.218	−0.039	0.207	0.086	0.228	0.075	0.221	0.107	0.222	0.117
QR	0.200	−0.065	0.208	0.137	0.189	0.051	0.194	0.074	0.195	0.083
AS	0.172	0.049	0.182	0.112	0.173	0.048	0.176	0.061	0.176	0.065
DID	0.605^a^	−0.118	0.614^a^	−0.745^a^	0.570^a^	−0.594^a^	0.563^a^	−0.673^a^	0.561^a^	−0.677^a^

Note

The loading value of considered morphological traits is shown for each species separately and for the two reduced subsets of *S. microphthalmus* as well. The following codes are the same showed in the dataset of Lunghi, Zhao, et al. (2019): Eye (eye diameter); Eye_ball (eyeball diameter); Snout (distance between the mouth tip and the beginning of the eye); Mouth width (length between the two mouth angles); Mouth length (length of the lower jaw); AD (linear distance between the snout tip and the top end of the head); B_height (head height measured at the nostril); C_height (head height measured at the eye); D_height (head height measured at the upper end); DI (linear distance between the top end of the head and the beginning of the dorsal fin); AE (maximum head length, measured from the snout tip until the farthest end of the head); FG (length of the forward pectoral fin base); IK (length of the dorsal fin base); I_depth (body depth measured at the beginning of the dorsal fin base); JW (length of the backward pectoral fin base); K_depth (body depth measured at the end of the dorsal fin base); NO (length of the anal fin base); O_depth (body depth measured at the end of the anal fin base); QR (caudal fin height at its base); AS (standard length); DID (humpback area). For each studied species we show the list of variables along their loading values for the first two PCA components.

NA means that the relative morphometric is not present for the species.

^a^ Factors for which we tested the divergence between populations (loading values ≥ 0.55).

### 3.2 *Sinocyclocheilus jii*

The PCA analysis grouped all populations into a single group (Figure 3b); the first two components together explained 93.16% of the variance (Table 1), and for both, the only important variable was the humpback area (Table 2). Although all specimens were grouped into a single group, specimens from Ji1 showed a significantly smaller humpback area compared to those from the other two populations (*df* = 2, *F* = 18.52, *p* < .001).

### 3.3 *Sinocyclocheilus microphthalmus*

**FIGURE 4:**
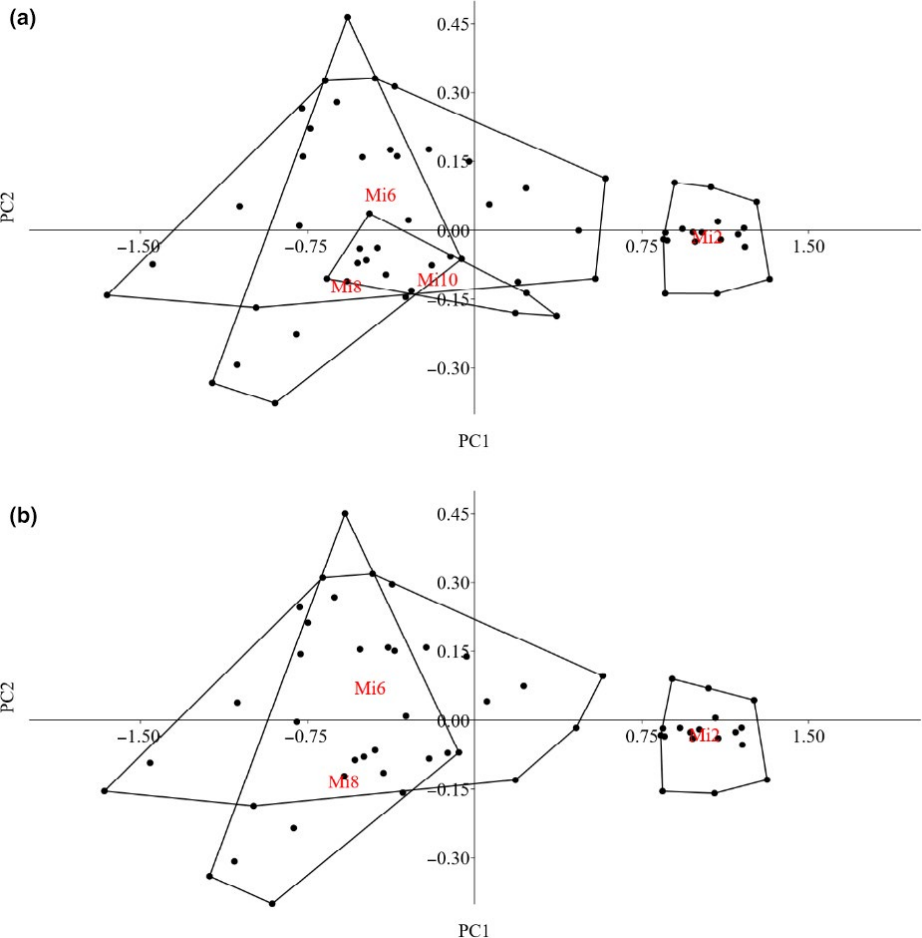
Principal component analysis of *Sinocyclocheilus microphthalmus* specimens accounting for season and year of collection. The PCA analysis is based on the same morphological features used in the main analysis. (a) Analysis only including specimens collected during the same season (March–July); (b) analysis only including specimens collected during 2019. The red labels within the convex polygons indicate the relative population

The PCA analysis identified two distinct groups, one including populations Mi2 and Mi5 and one with Mi6, Mi8, and Mi10 (Figure 3c); the first two components together explained 94.91% of the variance (Table 1). The most important variables for the principal components were the humpback area (PC1 and PC2) and the eyeball diameter (PC2) (Table 2). The two variables were significantly different between the two groups (eyeball diameter, *df* = 4, *F* = 8.36, *p* < .001; humpback area, *df* = 4, *F* = 63.39, *p < *.001); the group including Mi2 and Mi5 had specimens with bigger eye diameter and smaller humpback area. The results of the two additional PCA performed on different subsets of specimens were consistent with the previous ones, with the first two components explaining the majority of the variance (94.54% for the single season analysis and 95.09% for the single year). In both cases, the eyeball diameter showed a positive correlation with PC2, while the humpback area did show a positive correlation with PC1 and a negative with PC2. The Mi2 population was still clearly separated from the other group, which included Mi6, Mi8, and Mi10 in the analysis considering a single season (Figure 4a) and Mi6 and Mi8 in the analysis considering a single year (Figure 4b). Specimens from Mi2 showed significantly larger eyes (single season, *df* = 3, *F* = 8.53, *p < *.001; single year *df* = 2, *F* = 8.83, *p < *.001) and smaller humpback area (single season, *df* = 3, *F* = 46.5, *p < *.001; single year *df* = 2, *F* = 84, *p < *.001) compared to the populations included in the respective opposite group.

In both *S. brevibarbatus* and *S. microphthalmus*, there was a strong positive correlation between the eyeball diameter and the second principal component (PC2) (Table 2); in *S. jii* the correlation of this trait with both principal components was negligible (Table 2). In all three species, the humpback area was positively correlated with PC1 and negatively with PC2; however, for *S. brevibarbatus* the correlation with the PC2 was weak (Table 2).

## 4 DISCUSSION

With this study, we provided the evidence that intraspecific morphological variability occurs in *Sinocyclocheilus* cavefishes. In particular, in two out of the three studied species we observed a significant divergence for two morphological traits, namely the eyeball diameter and the humpback area (Table 2). According to our results, the first principal component likely represents the specimens' size, as all the considered morphological traits showed a positive correlation with this axis (Table 2). In the second principal component, some traits did show different correlation, particularly the eyeball diameter and the humpback area (Table 2), highlighting a potential different development according to the local ecological features (Christiansen, 2012; Howarth & Moldovan, 2018).

The populations of cavefish showing bigger eyeball diameter (*S. brevibarbatus*, Bb1 and *S. microphthalmus*, Mi2 and Mi5; Table 2, Figure 3a,c) likely inhabited areas not far from the connection with surface, where incoming light is still present, and thus, the use of sight is important (Culver & Pipan, 2014; Lunghi et al., 2015; Uiblein, 1992). Indeed, Mi2 specimens were collected from the cave entrance, while those belonging to populations Mi6, Mi8, and Mi10 were collected inside the cave more than 1 km far from the connection with surface, in areas laying in complete darkness. Unfortunately, no information on the collection site is available for Bb1, Bb2, and Mi5, and thus, future ecological surveys are needed to confirm this pattern. The small humpback area observed in Mi2 and Mi5 likely reflects a reduced need to store fat, probably as a consequence of a constant food availability (Culver & Pipan, 2014; Vandel, 1965); on the other hand, the increased humpback area observed in the conspecific Mi6, Mi8, and Mi10 populations likely indicate the need to store energy to better cope with irregular abundance of food resources (Culver & Pipan, 2019). The inconsistency of food resources in subterranean environments determines an oscillation of the humpback size, making it bigger during periods with high resource abundance and smaller when food resources are not available and cavefishes consume their own stored fat. Therefore, the divergence in the humpback area between specimens collected during different periods may not reflect a true intraspecific variability, but just provide information on different foraging histories. The analysis repeated with different subsets of *S. microphthalmus* confirms that a true variability occurs between individuals inhabiting shallow and deep groundwater habitats. In *S. brevibarbatus*, the correlation between the humpback area and the second principal component was negative, but very weak (Table 2). It may be possible that in this case, extraordinary abundance (for Bb1) and/or scarcity (for Bb2) of food resources occurred prior the collection time. This alternative hypothesis can be useful to discuss the results obtained for *S. jii* as well. Although the similarity in morphometrics shared by these specimens (no distinct groups were identified by the PCA analysis; Figure 3b), the humpback area of the population Ji1 was significantly smaller compared to the other two conspecific populations, likely meaning that such individuals experienced a shortage of food resources in the period before their collection.

This was the first study aiming to assess the intraspecific variability in Chinese cavefishes. We showed that morphological divergences in conspecific cavefish populations exist, and it likely occurs among the whole genus; however, future studies involving more species and individuals are needed to increase the generality of our findings. However, with our study we just scratched the tip of the iceberg, as we only focused on checking the morphological variability without investigating on their potential causes. Indeed, there are multiple factors responsible of this variability, such as adaptation, epigenetic effects, different time of colonization, and so on. Unfortunately, at the moment no information on the ecology and other life traits exists for these cavefish species, so we were unable to assess which are the potential drivers of the observed intraspecific morphological variability. It is not clear yet whether the similarities shared by cave‐adapted species are more likely caused by the resemblance of the experienced environmental conditions rather than phylogenetic contingency (Howarth, 2019); however, our study provided further information to shed light on this topic. The intraspecific variability observed in cavefishes highlighted a strong effect of the environment in promoting the development of specific traits, therefore supporting the hypothesis that species experiencing similar ecological conditions may develop similar traits (Howarth, 2019).

## CONFLICT OF INTERESTS

We declare no competing interests.

## AUTHOR CONTRIBUTION

**Enrico Lunghi:** Conceptualization (lead); Data curation (lead); Formal analysis (lead); Investigation (lead); Methodology (lead); Project administration (lead); Supervision (equal); Validation (equal); Visualization (lead); Writing‐original draft (lead); Writing‐review & editing (lead). **Yahui Zhao:** Funding acquisition (lead); Supervision (equal); Validation (equal); Writing‐review & editing (supporting).

## Supporting information

Appendix S1

## Data Availability

Data are available from the following publication: Lunghi, Zhao, et al. (2019).
