# Supplementary material for: Do Chinese cavefish show intraspecific variability in morphological traits?
Source: Ecol Evol. 2020 Jul 6;10(14):7723–30. doi: 10.1002/ece3.6495 (PMC7391565; doi:10.1002/ece3.6495)
Supplement: Supplementary file 1 — Appendix S1 [file ECE3-10-7723-s001.docx]

**Appendix**

Summary of the cavefishes’ measures provided in the dataset of Lunghi, Zhao, et al. (2019). Columns shows the data (average ± SD) for single *Sinocyclocheilus* species (in bold those analysed in this study), while each row corresponds to a specific cavefish body part (values for length are expressed in mm, while those for area in mm^2^). The following codes are the same showed in the dataset: Eye (eye diameter); Eye_ball (eye ball diameter); Snout (distance between the mouth tip and the beginning of the eye); Mouth width (length between the two mouth angles); Mouth length (length of the lower jaw); AD (linear distance between the snout tip and the top end of the head); B_height (head height measured at the nostril); C_height (head height measured at the eye); D_height (head height measured at the upper end); DI (linear distance between the top end of the head and the beginning of the dorsal fin); AE (maximum head length, measured from the snout tip until the farthest end of the head); FG (length of the forward pectoral fin base); FH (maximum extension of the forward pectoral fin); IM (maximum extension of the dorsal fin); IK (length of the dorsal fin base); I_depth (body depth measured at the beginning of the dorsal fin base); JL (maximum extension of the backward pectoral fin); JW (length of the backward pectoral fin base); K_depth (body depth measured at the end of the dorsal fin base); NO (length of the anal fin base); O_depth (body depth measured at the end of the anal fin base); NP (maximum extension of the anal fin); QR (caudal fin height at its base); QU (maximum extension of the top part of the caudal fin); ST (caudal fin mid length); RV (maximum extension of the low part of the caudal fin base); AS (standard length); AT (total length); DID (humpback area). Symbol. ”NA” means no data for the species.

|  | **Species** | | | | | | | | | | | | | | | |
| --- | --- | --- | --- | --- | --- | --- | --- | --- | --- | --- | --- | --- | --- | --- | --- | --- |
|  | ***S. brevibarbatus*** | | *S. brevis* | | *S. huanjiangensis* | | ***S. jii*** | | *S. lateristritus* | | *S. mashanensis* | | ***S. microphthalmus*** | | *S. qiubeiensis* | |
| **Body part** | Mean | SD | Mean | SD | Mean | SD | Mean | SD | Mean | SD | Mean | SD | Mean | SD | Mean | SD |
| Eye_diameter | 8.61 | 2.28 | 8.02 | 1.84 | NA | NA | NA | NA | NA | NA | 9.65 | 1.34 | 6.42 | 1.66 | NA | NA |
| Eye_ball | 3.42 | 0.70 | 3.83 | 0.71 | 4.20 | 1.02 | 4.38 | 0.84 | 5.77 | 0.99 | 3.65 | 0.41 | 1.48 | 0.51 | 5.66 | 0.52 |
| Snout_length | 11.13 | 2.80 | 6.99 | 2.37 | 6.55 | 2.39 | 6.82 | 1.94 | 9.25 | 2.80 | 13.45 | 2.36 | 9.61 | 3.06 | 10.61 | 1.40 |
| Mouth_width | 7.84 | 1.85 | 6.29 | 2.02 | 5.85 | 2.08 | 6.01 | 2.07 | 6.87 | 2.19 | 10.26 | 1.99 | 7.04 | 2.49 | 7.94 | 1.24 |
| Lower_jaw_length | 7.97 | 2.53 | 6.14 | 1.75 | 4.38 | 1.52 | 4.56 | 1.23 | 6.45 | 1.83 | 7.27 | 1.55 | 4.82 | 1.85 | 7.07 | 1.01 |
| AD | 25.19 | 5.08 | 18.37 | 5.20 | 15.82 | 5.20 | 15.91 | 3.96 | 20.71 | 5.27 | 29.70 | 4.82 | 22.54 | 6.31 | 22.91 | 1.99 |
| B_height | 8.77 | 1.68 | 8.55 | 2.13 | 6.58 | 2.34 | 6.65 | 1.99 | 8.47 | 2.39 | 10.44 | 1.95 | 6.71 | 2.25 | 8.82 | 1.35 |
| C_height | 13.08 | 2.65 | 10.89 | 3.29 | 9.44 | 3.20 | 9.82 | 2.47 | 12.32 | 3.15 | 15.06 | 2.14 | 9.45 | 3.24 | 13.36 | 1.54 |
| D_height | 19.00 | 3.87 | 15.85 | 4.96 | 13.07 | 4.67 | 13.66 | 3.48 | 17.17 | 4.35 | 22.30 | 2.74 | 16.41 | 5.16 | 18.99 | 1.91 |
| DI | 38.07 | 10.55 | 33.86 | 10.30 | 27.37 | 9.28 | 29.68 | 9.64 | 33.68 | 9.89 | 45.06 | 8.65 | 32.96 | 14.71 | 35.53 | 4.32 |
| AE | 33.94 | 7.48 | 24.07 | 7.25 | 20.63 | 7.02 | 21.47 | 5.79 | 27.00 | 6.84 | 40.84 | 6.86 | 28.67 | 8.62 | 30.19 | 3.73 |
| FG | 4.07 | 1.21 | 3.31 | 1.18 | 2.75 | 1.16 | 2.87 | 0.94 | 3.60 | 0.94 | 4.78 | 1.34 | 4.46 | 2.08 | 4 | 0.60 |
| FH | 27.44 | 6.08 | 20.81 | 7.30 | 14.60 | 6.28 | 15.64 | 4.79 | 17.73 | 4.99 | 33.34 | 6.12 | 27.34 | 12.53 | 19.41 | 2.56 |
| IM | 23.14 | 3.65 | 19.46 | 5.21 | 14.99 | 4.10 | 15.92 | 3.68 | 17.31 | 4.84 | 27.57 | 5.35 | 23.31 | 9.05 | 18.43 | 2.99 |
| IK | 16.08 | 3.59 | 12.95 | 3.87 | 9.95 | 3.13 | 9.56 | 2.99 | 12.02 | 3.41 | 18.60 | 3.81 | 16.32 | 7.90 | 13.89 | 2.59 |
| I_depth | 33.95 | 9.76 | 26.03 | 11.18 | 20.67 | 8.71 | 21.86 | 6.98 | 24.17 | 6.93 | 41.74 | 7.90 | 31.11 | 16.43 | 26.825 | 3.25 |
| JL | 19.96 | 3.33 | 15.57 | 4.59 | 12.23 | 4.10 | 13.35 | 3.45 | 14.59 | 4.13 | 24.42 | 5.12 | 20.49 | 8.41 | 14.85 | 1.64 |
| JW | 4.61 | 1.13 | 4.17 | 1.19 | 3.08 | 1.25 | 3.08 | 0.96 | 4.08 | 1.36 | 6.48 | 0.99 | 4.81 | 2.51 | 4.29 | 0.75 |
| K_depth | 24.67 | 7.09 | 21.02 | 8.40 | 16.93 | 7.01 | 16.99 | 5.65 | 18.41 | 5.53 | 31.07 | 6.03 | 23.53 | 12.01 | 20.92 | 2.63 |
| NO | 9.74 | 2.27 | 6.65 | 2.43 | 6.44 | 2.05 | 6.09 | 1.95 | 7.98 | 2.31 | 11.64 | 1.93 | 9.93 | 4.16 | 8.98 | 1.32 |
| O_depth | 15.20 | 4.22 | 14.22 | 4.55 | 11.20 | 4.27 | 11.72 | 3.97 | 13.17 | 4.13 | 18.77 | 3.79 | 14.37 | 7.35 | 14.35 | 1.48 |
| NP | 18.95 | 3.48 | 14.73 | 4.16 | 11.93 | 4.42 | 13.07 | 3.17 | 15.18 | 4.25 | 23.70 | 3.08 | 19.28 | 6.88 | 15.42 | 2.03 |
| QR | 15.00 | 3.70 | 12.37 | 3.93 | 10.69 | 3.67 | 10.60 | 3.43 | 11.86 | 3.41 | 16.99 | 3.00 | 13.44 | 5.41 | 12.45 | 1.42 |
| QU | 29.76 | 5.17 | 26.30 | 5.35 | 20.97 | 4.55 | 21.07 | 4.83 | 23.34 | 6.67 | 34.69 | 5.49 | 28.96 | 10.03 | 26.71 | 2.88 |
| ST | 13.08 | 2.36 | 12.34 | 3.08 | 10.71 | 2.99 | 10.76 | 2.95 | 13.49 | 3.89 | 14.75 | 2.75 | 12.33 | 4.89 | 14.09 | 1.63 |
| RV | 31.81 | 4.86 | 27.10 | 6.61 | 20.18 | 4.80 | 21.89 | 4.63 | 24.29 | 6.16 | 33.71 | 5.64 | 29.21 | 10.80 | 26.705 | 2.36 |
| AS | 110.04 | 24.12 | 93.02 | 26.52 | 73.75 | 25.87 | 80.65 | 23.43 | 90.97 | 25.84 | 130.85 | 21.37 | 104.20 | 37.83 | 102.64 | 10.69 |
| AT | 125.69 | 26.57 | 106.11 | 28.75 | 84.24 | 26.05 | 91.02 | 26.31 | 106.25 | 29.73 | 145.72 | 24.52 | 117.33 | 41.90 | 116.4 | 11.88 |
| DID | 106.41 | 117.53 | 62.24 | 52.90 | 38.09 | 26.56 | 43.16 | 54.12 | 38.65 | 26.33 | 175.09 | 84.14 | 53.92 | 113.24 | 29.14 | 18.51 |
